# Benefit assessment of extended dosing in cancer patients after their withdrawal from clinical trials

**DOI:** 10.3389/fphar.2023.1178002

**Published:** 2023-12-15

**Authors:** Feng Yang, Zhe Huang, Jianfu Heng, Kunyan Li

**Affiliations:** ^1^ Hunan Cancer Hospital, Xiangya School of Medicine, Central South University, Changsha, Hunan Province, China; ^2^ Department of Pathology, Immuno-Oncology Laboratory, School of Basic Medicine, Central South University, Changsha, Hunan, China

**Keywords:** clinical trials, withdrawal, extended dosing, economic cost, overall survival

## Abstract

**Background:** Clinical trials have been widely recognized as an effective treatment approach by physicians and cancer patients alike. Physicians’ evaluations suggest that many patients are likely to continue experiencing benefits from extended dosing of investigational new drugs even after withdrawing from clinical trials.

**Objective:** Given the uncertainty surrounding the efficacy and safety of investigational new drugs, it is essential to continually assess the benefits of extended dosing for patients.

**Methods:** The trial group for this study comprised patients who requested extended dosing after withdrawing from clinical trials at Hunan Cancer Hospital between 2016 and 2020. The control group consisted of patients who received conventional treatment and were enrolled in a 1:1 ratio. Follow-up assessments were conducted every 3 months for both groups, and included monitoring of patients’ health status, survival time, disease control or remission, treatment modalities received, and medical costs.

**Results:** A total of twenty-three patient pairs were successfully matched for this study. The Ethics Committee approved extended dosing for all patients in the trial group, with an average gap period of 16.48 days between their withdrawal from clinical trials and continuous access to the investigational drugs. The median overall survival for patients after withdrawal from clinical trials was 17.3 months in the extended dosing group and 12.9 months in the control group, with no significant difference observed between the two groups (*p* > 0.250). The median total cost of treatment after the previous clinical trial was 38,006.76 RMB, of which the median cost of therapeutic drugs for conventional treatment was 15,720 RMB, while extended dosing was provided free of charge.

**Conclusion:** Extended dosing can indeed provide benefits, including survival benefits and economic benefits, to cancer patients after their withdrawal from clinical trials and will clinically present an additional treatment option for patients.

## Introduction

Cancer is the major public health problem in the world (Miller et al., 2022). Participating clinical trials has already been considered as an effective way of cancer therapy by doctors and patients (Li et al., 2019). However, 70% people tend to or are more willing to participate in clinical trials, only around 5% cancer adults finally did. One of the main reasons is the limitation of rigorous inclusion and exclusion standards in the clinical trial protocols (Unger et al., 2016).

Expanded compassionate use, also known as expanded access, offers a means of accelerating patient access to investigational new drugs for rare and severe diseases when patients have not been recruited or are not eligible for recruitment in clinical trials (Jommi et al., 2021). The Food and Drug Administration encourages drug sponsors to provide expanded access to investigational drugs to patients who are unable to enroll in a clinical trial, when feasible and warranted (Darrow et al., 2015). While in China, the General Office of the CPC Central Committee and the State Council issued Opinion about Deepening the reform of the review and approval System to encourage innovation of drugs and medical devices in 2017 (The General Office of the Central Committee of the Communist Party of China and the General Office of the State Council, 2017), which supports expanded clinical trial policies. In the end of the same year, former China Food and Drug Administration drafted and published Management Methods of Expanded Compassionate Use of Clinical Trial Drugs (draft for advice) (General Office of China Food and Drug Administration, 2017), but it has not been officially implemented yet. Clause 23 in the newly revised The Drug Administration Law of the PRC (NPC’s standing committee of China, 2019) in 2019 officially defined the applicable conditions and procedures of compassionate use.

Clinical trials offer an essential form of therapy for patients who gain access to investigational new drugs (Zhou et al., 2017). To ensure the safety of participants and the accuracy of the study results, strict withdrawal criteria are put in place for every new drug trial. Patients who exhibit disease progression or abnormal intolerance to adverse events may be withdrawn from the trial (Ulrich et al., 2021). To the best of our knowledge, some patients may experience positive health outcomes after being withdrawn from clinical trials, either because they did not developed any significant tumor progression or did not face any irreversible safety risk. In such cases, it is generally considered ethical to arrange post-trial care for these patients (Cho et al., 2018). Investigators and sponsors have a responsibility to continuously provide investigational new drugs to patients probably benefiting from trial drugs after their withdrawal from trials, which is called “extended dosing.”

Prior to implementing extended dosing, it is required to obtain approval from the Ethics Committee (Borysowski et al., 2017), and our hospital took the lead in carrying out the exploration and practice of extended dosing in 2016. Currently, ethical review of extended dosing is typically focused on the initial review, and there is a lack of systematic ethical follow-up review during the implementation of extended dosing. It is uncertain whether the patients ultimately benefit from extended dosing. Our study aims to reassess the risks and benefits of patients during the implementation of extended dosing, and the results will help standardize the implementation of extended dosing and the development of ethical guidance.

## Methods

### Patients

A total of 1,918 patients had been withdrawn from clinical trials between 2016 and 2020, and 33 patients submitted requests for extended dosing to the Ethics Committee. The trial group consisted of patients who submitted requests for extended dosing after withdrawal from clinical trials. Each patient in the control group originated from the same clinical trial as the patients in the trial group. Due to the limited number of enrolled patients, we utilized a 1:1 matching method to ensure the basic characteristics of both patient groups were comparable. As a result, the treatment conditions were generally similar. The inclusion criteria for both groups were provided below, while no exclusion criteria were specified.

### Inclusion criteria of trial group

(1) Patients who had completed the registered new drug clinical trials as per the protocol or who had been discharged from the clinical trials according to the protocol withdrawal criteria.
(2) The investigators deemed that the benefits of continuing medication outweighed the risks.
(3) The sponsors agreed to continue providing the investigational new drugs.
(4) Patients had given full informed consent to continue using the investigational new drugs.

### Inclusion criteria of control group

(1) The group in which patients were originally assigned during the previous clinical trial should be the same group to which they are assigned in the extended dosing study.
(2) The reasons for withdrawal from the previous clinical trials should be the same as those for the patients participating in the extended dosing study.
(3) The general characteristics of the patients, including their smoking history, age, ECOG PS score, and disease stage, were similar to those of the trial group.

### Treatment

Both groups were followed up through telephone or outpatient visits every 3 months until death or until they were followed up for more than 1 year, whichever occurred first. During these follow-up visits, patients in the trial group continued to receive the investigational drugs until the investigators determined that no further benefit could be achieved, or the patients voluntarily discontinued extended dosing. The control group received conventional therapy after withdrawing from the previous clinical trials. Neither group received any additional study interventions. The last follow-up was conducted in August 2022, and it included assessments of the patients’ health status, survival time, disease control or remission, treatment modalities received, and medical costs.

### End points

The efficacy of extended dosing was evaluated based on overall survival (OS) from the time of patients’ withdrawal from the previous clinical trials to death from any cause. For some patients who were lost to follow-up prior to death, the last follow-up was counted as the time of death. To assess the long-term benefit of extended dosing, the OS of the two groups was compared. In addition, an economic benefit analysis was conducted to evaluate the cost of therapeutic drugs after the patients’ withdrawal from the previous clinical trials.

### Statistical analyses

Unless otherwise specified, the data in this study will be summarized using the following general principles: the Kaplan-Meier method will be utilized to estimate the survival function for time-event data, and survival curves will be plotted to estimate the median time and its 95% confidence interval. Differences between groups were compared by Log-rank test. Categorical data will be descriptive by frequency n and percentage % and compared by Chi-square test or Fisher’s exact test. Bilateral 0.05 level test was used for all statistical tests, and *p* < 0.05 was considered statistically significant. The clinical database was locked on 21 August 2022. Microsoft Excel 2016 and SPSS version 25.0.0 were used for statistical analysis and reporting of the data collected for this study.

## Results

### Available data sets

Between 2016 and 2020, 33 patients (1.72%) voluntarily submitted an application for extended dosing of the investigational drug after withdrawing from clinical trials. These applications were accompanied by a risk-benefit assessment by the investigators. After undergoing full review by the Ethics Committee, 31 cases of extended dosing were approved, while 2 cases were disapproved. The main reasons for the disapproval of these two cases were that the patients who applied for extended dosing had developed secondary disease progression and were experiencing grade 4 adverse events, and that the sponsor expressed reluctance to continue providing the trial drugs for the patients and was unwilling to bear the associated risks.

**FIGURE 1:**
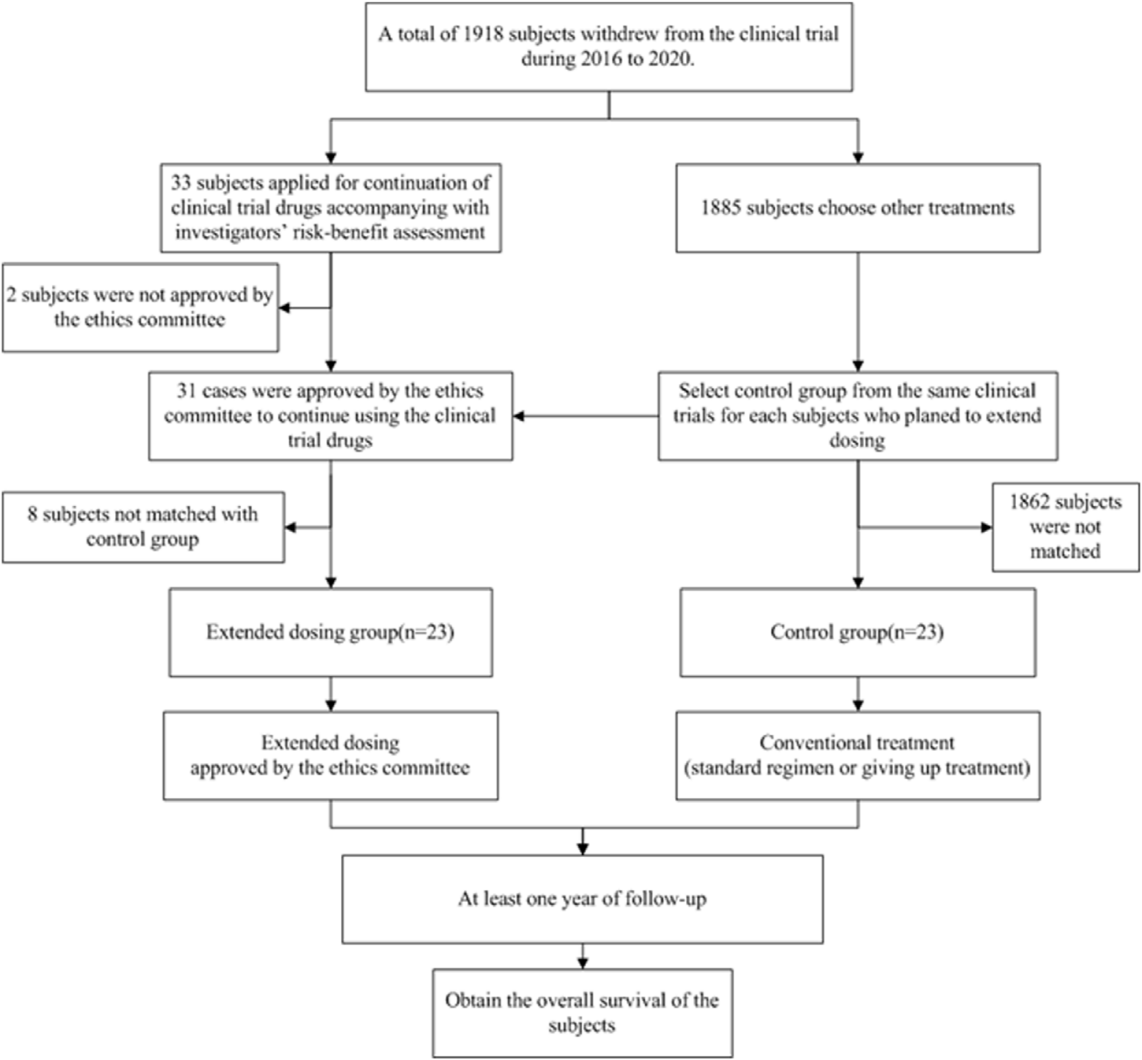
Flow chart of study design.

The control group was matched at a 1:1 ratio, and 23 pairs of patients were successfully matched, while 8 patients who received extended dosing were not matched with the control group due to insufficient population or not meeting the main matching conditions, such as inconsistent withdrawal reasons (Figure 1). The median length of participation in the previous clinical trials was 90 days, and the average duration of extended dosing was 62 days. At the last follow-up, one patient was still receiving extended dosing treatment (Supplementary Table S1).

### Clinical trial characteristics

Among the patients in the extended dosing group, 10 (43.5%) patients participated in the phase I clinical trial, 17 (73.9%) patients received targeted therapy in the clinical trials. In addition, the majority of patients in the extended dosing group had lung cancer as their primary tumor site (69.6%) and were classified as receiving posterior line therapy (78.3%) in the clinical trials (Table 1).

**TABLE 1:** Characteristics of clinical trails included (*n* = 23).

Characteristics	Number of cases (%)
Clinical Trial Phase	
I	10 (43.5)
II	8 (34.8)
III	5 (21.7)
Type of treatment	
Target therapy	17 (73.9)
Immunotherapy	3 (13.0)
Antiangiogenic therapy	3 (13.0)
Primary tumor cite	
Lung cancer	16 (69.6)
Liver cancer	2 (8.7)
Lymphoma	1 (4.3)
Ovarian cancer	1 (4.3)
Prostate cancer	2 (8.7)
Breast cancer	1 (4.3)
Treat lines	
First line	5 (21.7)
post line	18 (78.3)

### Patient characteristics

The median age of the total population was 56 years (range 30–75 years), with 28 (60.8%) male patients. Of the total population, 18 (60.8%) were smokers, 45 (97.8%) had an ECOG score of 0–1, 35 (76.1%) had no brain metastases, and 37 (80.4%) had disease progression. There were no statistically significant differences in baseline characteristics between the two groups (*p* < 0.05) (Table 2).

**TABLE 2:** Patients clinical characteristics.

Characteristics	Extended dosing group (*n* =23)	Control group (*n* =23)	*p*-value
Age (year, middle, range)	57(40–75)	57(30–71)	0.965
Gender			0.365
Male	12 (52.2)	16 (69.6)	
Female	11 (47.8)	7 (30.4)	
Smoking history			0.365
Smoker	7 (30.4)	11 (47.8)	
Never smoked	16 (69.6)	12 (52.2)	
ECOG PS			1.000
Good ( 0-1)	23 (100)	22 (95.7)	
Poor (2-3)	0 (0)	1 (4.3)	
Brain metastasis			1.000
With	6 (26.1)	5 (21.7)	
Without	17 (73.9)	18 (78.3)	
Reason for clinical trial termination			1.000
Disease progression	18 (78.3)	19 (82.6)	
Adverse event	3 (13.0)	3 (13.0)	
Meet exclusion criteria	1 (4.3)	0	
Start a new anti-tumor therapy	1 (4.3)	1 (4.3)	

### Efficacy

**FIGURE 2:**
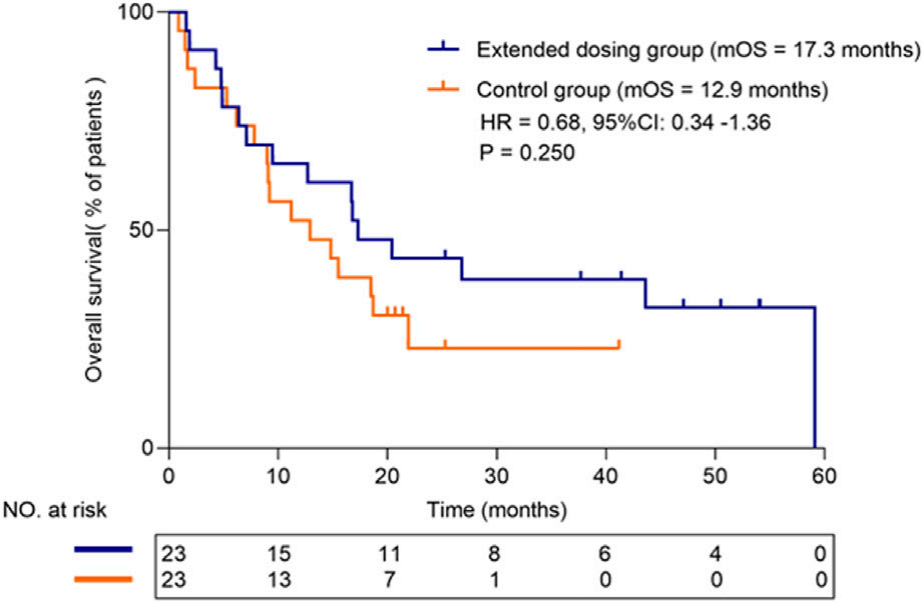
Survival plots of overall survival for treated and control groups.

The median follow-up time until the last follow-up was 41.2 months (95% CI: 34.6–47.8 months). The median OS was 17.3 months (95% CI: 12.4–22.2 months) in the extended dosing group and 12.9 months (95% CI: 9.1–16.7 months) in the control group. There was no significant difference in OS between the two groups (HR = 0.68, 95% CI: 0.34–1.36, *p* = 0.250) (Figure 2).

### Economic benefit

Patients in the extended dosing group received the investigational drugs therapy free of charge, while they had to pay for the standard of care, laboratory examinations, combination therapy, and diagnostic procedures. In contrast, patients in the control group had to bear all cancer treatment-related expenses, including the cost of therapeutic drugs.

Among the 23 patients in control group, two patients participated in other antitumor drug clinical trials to continue their antitumor therapy after withdrawing from the previous clinical trials. Additionally, four patients abandoned treatment, and two patients received traditional Chinese medicine for their treatment.

**FIGURE 3:**
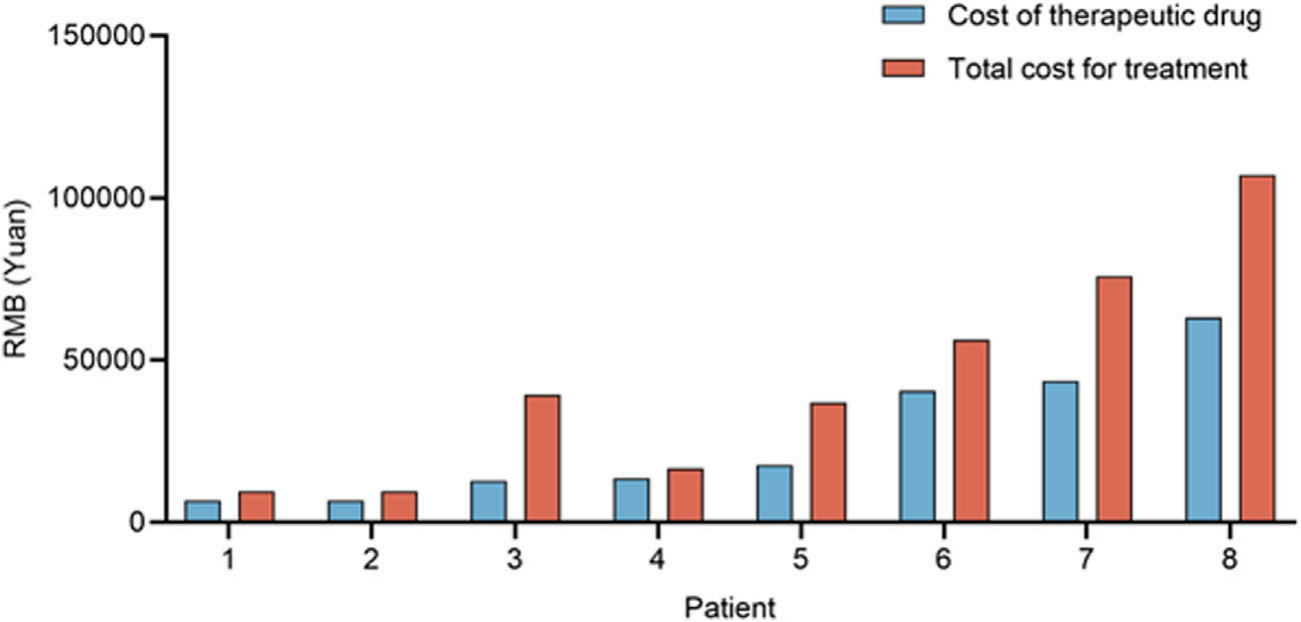
Cost of first-line therapy after withdrawal for 8 patients in the control group.

Fifteen patients had started a new regimen of antitumor therapy, but seven of them received treatment at a local hospital. We were able to access the medication records of eight patients, and after analyzing their total treatment costs, the median cost for treatment after the previous clinical trial was found to be 38,006.76 RMB. The median cost of therapeutic drugs was 15,720 RMB, as shown in Figure 3.

### Gap period

The consideration period refers to the interval between patients’ withdrawal from clinical trials and their voluntary application for extended dosing, which is accompanied by a risk-benefit assessment by investigators. The approval period denotes the duration between patients’ application for extended dosing, along with the investigators’ risk-benefit assessment, and the subsequent ethical approval. The gap period refers to the time between withdrawing from the clinical trial and continuing with extended dosing. Considering that the control group did not have a process, we only analyzed it in the trial group.

Out of the 23 patients who applied for extended dosing, four patients commenced the treatment without ethical approval, but it was eventually granted by the Ethics Committee. The average duration of the consideration period was 10.13 days, while the average approval period was 12.13 days. The average gap period between withdrawing from the clinical trial and starting the extended dosing was 16.48 days, as shown in Table 3.

**TABLE 3:** Analysis of time spent at different stages of extension dosing.

Patient group	Duration, median (IQR), days		
	Patients’ withdrawal from clinical trials to application for extended dosing	Patients’ application for extended dosing to ethical approval	Patients’ withdrawal from clinical trials to continuation of extended dosing
Exteded dosing	2 (0–9)	7 (4–12)	11 (5–27)

## Discussion

As is widely known, the probability of a new drug entering clinical trials and eventually being approved for sale is very low (Borysowski and Gorski, 2019). However, investigators must still present clinical trials as providing the “prospect of a direct medical benefit,” particularly in oncology, where the boundaries between clinical research and treatment are often blurred (Gerasimov et al., 2020). As the American Society of Clinical Oncology has stated, phase I clinical trials are a treatment modality with potential clinical benefits for patients with advanced-stage malignancies (Howard et al., 2021). The moment of clinical trial withdrawal is crucial for patients and their families, who have invested time and hope in the trials. For cancer patients facing life-threatening and life-limiting illnesses, what happens at trial exit is just as significant as what happens at entry (Martinez, 2019; Ulrich et al., 2021). Extended dosing presents a new treatment option for patients who have withdrawn from trials. Based on our analysis, extended dosing can offer patients both efficacy and economic benefits. On one hand, the extended dosing group had a median overall survival time 4.4 months longer than that of the conventional treatment group after patients’ withdrawal from clinical trials. On the other hand, the medication cost of the next-line treatment for the extended dosing group after trial withdrawal was 15,720 RMB less than that of the conventional treatment group. The analysis of clinical data from the 23 patients who continued to receive investigational new drug interventions represents one of the first investigations into the efficacy of extended dosing in the field of oncology.

However, which types of cancer patients are eligible for extended dosing? Based on our analysis of clinical data, there may be four categories. Firstly, patients with disease progression in non-target lesions but well-controlled in target lesions and with no unacceptable or irreversible safety risk after withdrawing from clinical trials may be considered for extended dosing. Secondly, patients whose tumors have progressed but had previously shown good control, and who currently exhibit mild or no clinical symptoms with a significantly improved quality of life, may also be eligible. In these circumstances, patients may qualify for extended dosing. Thirdly, enrolled patients who were subsequently found to have violated the eligibility criteria but were effectively treated in trials may have been able to continue receiving extension therapy. Abruptly discontinuing treatment with new drugs may be unethical in such cases. Fourthly, some participants in the low dose group of phase I dose-escalation trials may apply for extension treatment with higher doses, as their plasma concentrations may not reach the effective concentration. From the above discussion, we can conclude that the clinical needs of the patient’s disease, the safety benefit, and the effectiveness benefit should be the main factors considered when assessing whether a patient is suitable for extended dosing.

One of the main findings of this study is that the median overall survival of patients receiving extended dosing is 4.4 months longer than that of patients receiving conventional treatment after withdrawal from clinical trials, indicating that extended dosing does not result in inferior overall survival compared to conventional therapy. As shown in previous studies by Qing Chang et al., for patients with epidermal growth factor receptor (EGFR) mutations who progressed gradually after initial tyrosine kinase inhibitor (TKI) therapy but with no EGFR-T790M mutation, continued oral TKI therapy was still beneficial in progression-free survival compared with conventional chemotherapy (Chang et al., 2021). Similarly, according to Yaxiong Zhang et al.'s study (Zhang et al., 2017), patients in the L858R cohort with gradually progressive disease would gain benefits from continuing their prior TKI therapy. Additionally, patients with oligoprogression and no significant disease progression may benefit from local treatment alongside their current treatment regimen, such as extended dosing. In a study conducted by Xu Q et al. (Spry et al., 2018), the addition of local ablation therapy to EGFR-TKI demonstrated a satisfactory survival benefit for patients with EGFR-mutated NSCLC who experienced oligoprogression during their first-line EGFR-TKI therapy (Jiang et al., 2019). Similarly, radiotherapy has the potential to improve progression-free survival (PFS) and prolong the efficacy of immune checkpoint inhibitors in patients experiencing oligoprogression (Sindhu et al., 2022). Similar to our findings, these studies indicate that in certain scenarios, patients with advanced disease may still respond well to their previous treatment regimen. Thus, extended dosing can indeed provide survival benefits for patients after discontinuing their participation in clinical trials.

It is also worth mentioning that extended dosing can greatly reduce the financial burden of posterior line antitumor therapy for patients who exit clinical trials. In our practice, trial drugs were provided to patients at no cost, whereas conventional treatment after trial withdrawal had a median cost of 15.7 thousand RMB for therapeutic drugs. Currently, there are no established guidelines in China for the administration of extended dosing, and no regulations on the cost of extended dosing drugs. In the absence of such guidelines, sponsors have taken the voluntary and responsible approach of providing investigational drugs to patients who may still derive benefits. This approach aligns with the principles of Good Clinical Practice regulations and ethical guidelines, and serves to safeguard the rights and interests of patients. In most cases, investigational drugs are provided at no cost to patients during the extended dosing phase. However, in the United States, sponsors may be allowed to charge patients for drugs provided under expanded-access programs as required by the FDA (21 CFR 312.8). In the European Union, France has implemented a rule that permits sponsors to request support fees from the country (Tsuyuki et al., 2016). The responsibility for covering the expenses of an unapproved product in the global market remains ambiguous. However, in China, extended dosing could potentially alleviate the financial burden on patients, enabling them to allocate their resources towards other medical examinations or treatments for combined symptoms, thus providing significant financial benefits.

Despite our best efforts, our study had several limitations. Firstly, the sample size was small, which may limit the generalizability of our findings. Additionally, patients in the same group had different types of cancer and received various treatments, which could have introduced bias in the statistical analysis of overall survival. Thirdly, we were unable to analyze all subsequent treatments for patients and determine the cost-effectiveness of the intervention after patients withdrew from the trial. Finally, the safety of extended dosing patients was not fully evaluated, which is an important consideration for future research.

## Conclusion

To conclude, extended dosing could potentially offer benefits to patients with severe life-threatening illnesses who have withdrawn from clinical trials, thus providing them with an additional treatment option.

## Data Availability

The original contributions presented in the study are included in the article/Supplementary Material, further inquiries can be directed to the corresponding author.
