# Supplementary material for: Benefit assessment of extended dosing in cancer patients after their withdrawal from clinical trials
Source: Front Pharmacol. 2023 Dec 15;14:1178002. doi: 10.3389/fphar.2023.1178002 (PMC10757887; doi:10.3389/fphar.2023.1178002)
Supplement: Supplementary file 1 [file Table1.docx]

**Table S1. The enrollment and withdrawal reasons for 23 patients**

| Patient | Time in clinical trails (d) | Reason for extended dosing | Time for extended dosing (d) | Reason for ending extended dosing |
| --- | --- | --- | --- | --- |
| P1 | 36 | Investigator assessed continued benefit | 273 | developed disease progression again |
| P2 | 21 | Poor patient income | 744 | continue extended dosing |
| P3 | 113 | Phase I clinical trial, increasing dose may continue to benefit | 28 | developed disease progression again |
| P4 | 132 | Investigator assessed continued benefit | 126 | developed disease progression for the third time |
| P5 | 43 | Investigator assessed continued benefit | 52 | developed disease progression again |
| P6 | 8 | The drug used outside the patient 's protocol had no antitumor effect and was intended to be continued | 21 | showed intolerable adverse events |
| P7 | 1 | Chemotherapy drug allergy, changed chemotherapy drug to continue medication | 71 | developed disease progression again |
| P8 | 357 | Investigator assessed continued benefit | 55 | The investigator concluded that patient would no longer benefit from continuing using new drug |
| P9 | 90 | Investigator assessed continued benefit | 13 | developed disease progression again |
| P10 | 32 | Investigator assessed continued benefit | 218 | Patient self-terminating |
| P11 | 42 | Investigator assessed continued benefit | 83 | developed disease progression again |
| P12 | 34 | Investigator assessed continued benefit | 308 | developed disease progression again |
| P13 | 175 | Investigator assessed continued benefit | 41 | developed disease progression again |
| P14 | 293 | Investigator assessed continued benefit | 58 | developed disease progression again |
| P15 | 126 | Investigator assessed continued benefit | 51 | Death |
| P16 | 120 | Investigator assessed continued benefit | 174 | developed disease progression again |
| P17 | 69 | Investigator assessed continued benefit | 62 | started a new anti-tumor therapy |
| P18 | 168 | Investigator assessed continued benefit | 513 | started a new anti-tumor therapy |
| P19 | 51 | Investigator assessed continued benefit | 382 | developed disease progression again |
| P20 | 194 | Investigator assessed continued benefit | 31 | developed disease progression again |
| P21 | 8 | Adverse event returned to normal and medication was continued | 21 | showed intolerable adverse events |
| P22 | 214 | Investigator assessed continued benefit | 62 | developed disease progression again |
| P23 | 337 | Investigator assessed continued benefit | 1 | started a new anti-tumor therapy |
| The median value | 90 |  | 62 |  |
