# Supplementary figures and images for: Benefit assessment of extended dosing in cancer patients after their withdrawal from clinical trials
Source: Front Pharmacol. 2023 Dec 15;14:1178002. doi: 10.3389/fphar.2023.1178002 (PMC10757887; doi:10.3389/fphar.2023.1178002)

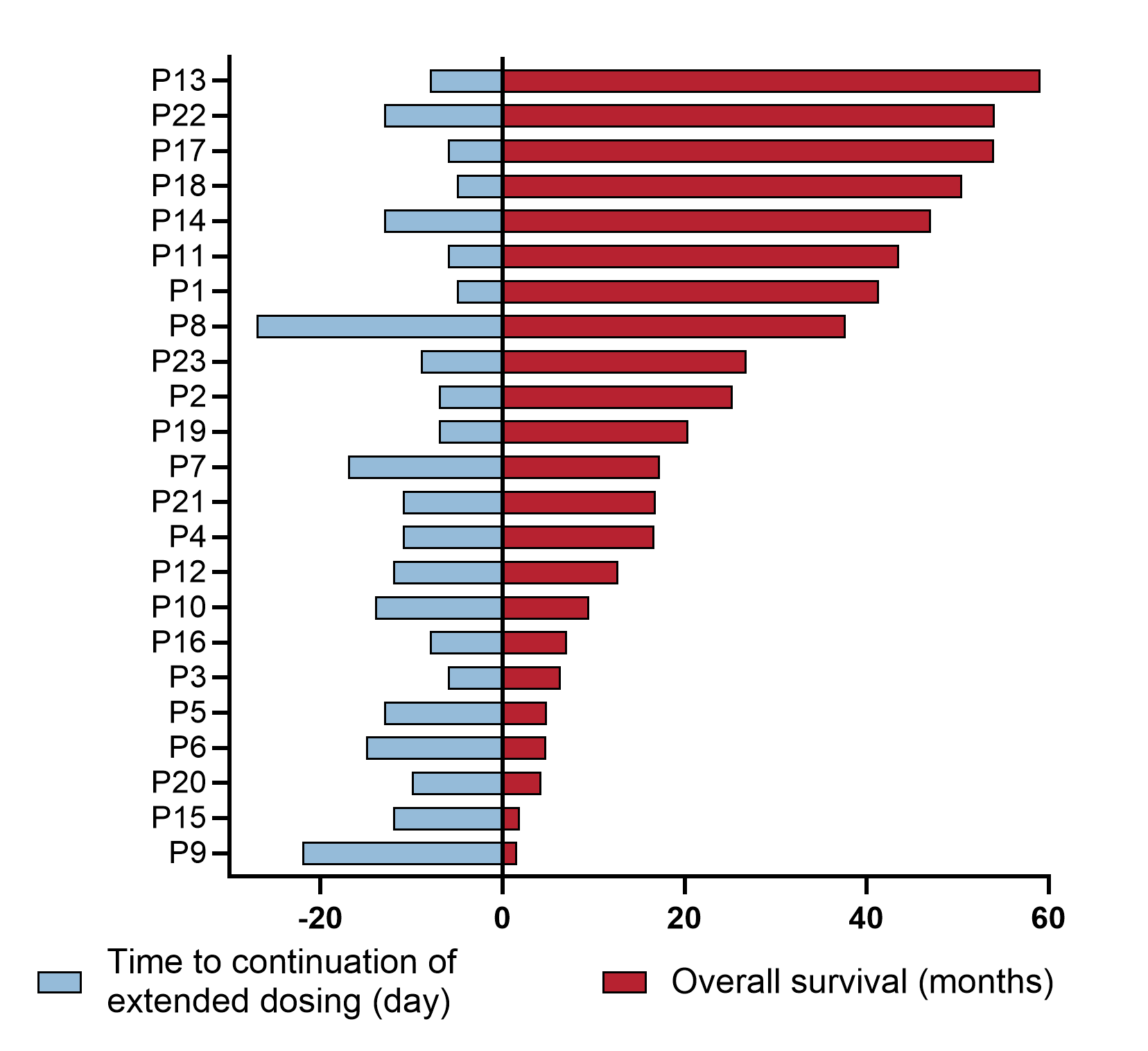

Supplement: Supplementary file 2 [file Image1.TIF]
